# When Guidelines Fail: A Case Report of Rule-Negative Traumatic Subarachnoid Hemorrhage From a Contrecoup Mechanism

**DOI:** 10.7759/cureus.95378

**Published:** 2025-10-25

**Authors:** Ahmed Ahmed, Omar Elsayed, Adeem Qamar, Oussama Medjahed, Dianne Tabone

**Affiliations:** 1 Emergency Medicine, Royal Preston Hospital, Lancashire Teaching Hospitals NHS Foundation Trust (LTHTR), Preston, GBR; 2 General Practice, Royal Preston Hospital, Lancashire Teaching Hospitals NHS Foundation Trust (LTHTR), Preston, GBR; 3 Critical Care, Royal Preston Hospital, Lancashire Teaching Hospitals NHS Foundation Trust (LTHTR), Preston, GBR

**Keywords:** clinical decision rules, computed tomography (ct ), emergency medicine and trauma, neurosurgery, nice guidelines, rule-negative, traumatic brain injury, traumatic subarachnoid hemorrhage

## Abstract

Subarachnoid hemorrhage (SAH) is a serious neurological emergency most commonly caused by aneurysmal rupture or major trauma. Clinical decision rules are widely applied to determine the need for early CT imaging following head trauma or loss of consciousness. We present the case of a 30-year-old man who collapsed at home with a brief loss of consciousness and nonspecific symptoms. Although he did not meet established CT head criteria, imaging was performed due to persistent clinical concern. This demonstrated an SAH and a right-sided frontal contusion. CT angiography excluded an aneurysm or vascular malformation. The hemorrhage distribution was consistent with a contrecoup mechanism secondary to occipital impact. Contrecoup-related SAH is exceedingly rare, with only a handful of isolated cases reported in the literature. The patient remained neurologically stable and was discharged without deficits after inpatient observation, with complete recovery at follow-up. This case highlights that significant intracranial pathology may occur in patients who do not meet standard criteria, underscoring the importance of clinical judgment alongside guidelines. Furthermore, while contrecoup injuries are relatively frequent, it is uncommon for them to cause SAH with a distribution mimicking aneurysmal rupture, emphasizing the importance of correlating clinical suspicion with imaging findings.

## Introduction

Subarachnoid hemorrhage (SAH) is a life-threatening neurological condition, most often due to ruptured intracranial aneurysms or severe trauma. Clinical decision rules such as the National Institute for Health and Care Excellence (NICE) Head Injury Guidelines [1] and the Canadian CT Head Rule [2] are frequently used to guide imaging decisions, balancing diagnostic accuracy with resource use. However, these rules are not flawless, and significant pathology can occasionally be missed if decisions rely solely on them.

Traumatic SAH accounts for approximately 30%-60% of moderate to severe head injuries, but cases attributed specifically to a contrecoup mechanism are exceptionally uncommon, described almost exclusively in isolated case reports [3-5]. A contrecoup injury occurs when rapid deceleration or impact causes the brain to strike the skull opposite the site of impact, leading to vascular tearing and parenchymal or subarachnoid bleeding on the contralateral side.

Contrecoup injuries are a recognized mechanism in traumatic brain injury, typically causing frontal or temporal contusions opposite the site of impact [3-5]. Rarely, they may lead to SAH, and in some cases, the bleeding pattern may resemble spontaneous aneurysmal SAH [4,5]. To date, only a handful of contrecoup-related SAH cases have been documented in the literature, most describing basal or frontal bleeding following occipital impact, underscoring the exceptional rarity of this mechanism. However, such cases may be underrecognized when patients do not meet established CT head criteria-highlighting a gap in guideline sensitivity and the need for heightened clinical vigilance. We report a case of rule-negative traumatic SAH in a young adult male patient, with imaging findings suggestive of a contrecoup mechanism.

## Case presentation

A 30-year-old male patient with no significant past medical history presented to the emergency department following a transient loss of consciousness at home. He had woken at 5 a.m. to use the bathroom, and during this time, he felt lightheaded, weak, and dizzy. Shortly afterward, he collapsed and was found on the floor by his wife. There was no witnessed seizure activity or incontinence. Loss of consciousness lasted less than five minutes. Upon regaining consciousness, he complained of a persistent headache and nausea. The collapse was initially thought to represent a vasovagal or micturition syncope with secondary trauma on impact; however, the absence of recurrent episodes, bradycardia, or hypotension made this less likely.

There was no history of chest pain, palpitations, vomiting, or shortness of breath. He denied illicit drug use. He reported several days of loose stools and malaise before the event, but no neurological symptoms. These were mild, and there were no clinical or biochemical features of dehydration or electrolyte imbalance, making them unlikely to have contributed to the syncopal episode. He was not taking anticoagulants.

On arrival, his Glasgow Coma Scale (GCS) score was 15. Neurological examination was normal with no focal deficits. Vital signs were stable and within normal limits, and all investigations, including blood tests and ECG, were unremarkable.

**Figure 1:**
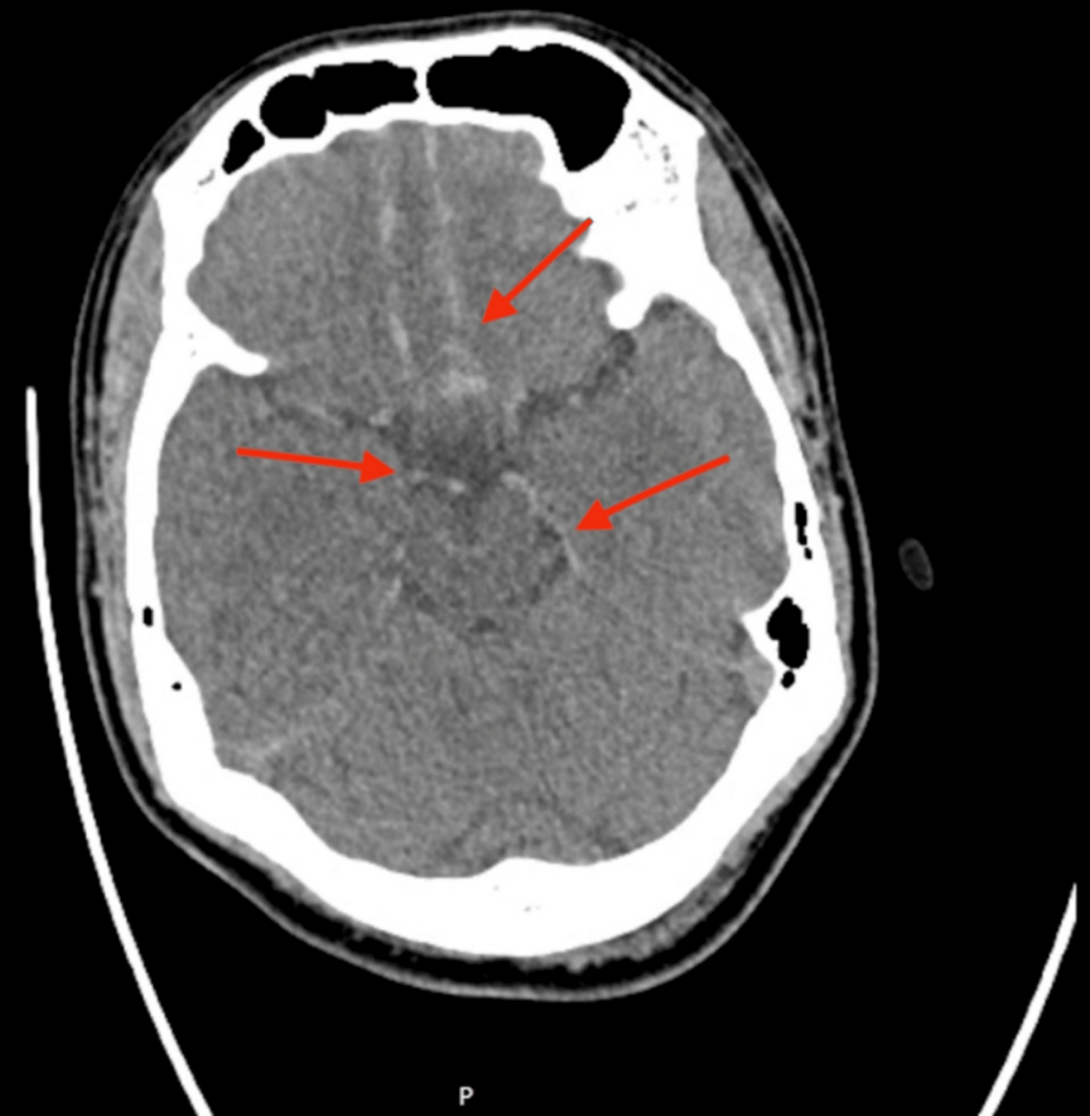
Subarachnoid hemorrhage Axial noncontrast CT brain demonstrating acute subarachnoid hemorrhage within the suprasellar cistern, extending over the inferior frontal cortex and along the falx cerebri (red arrows)

**Figure 2:**
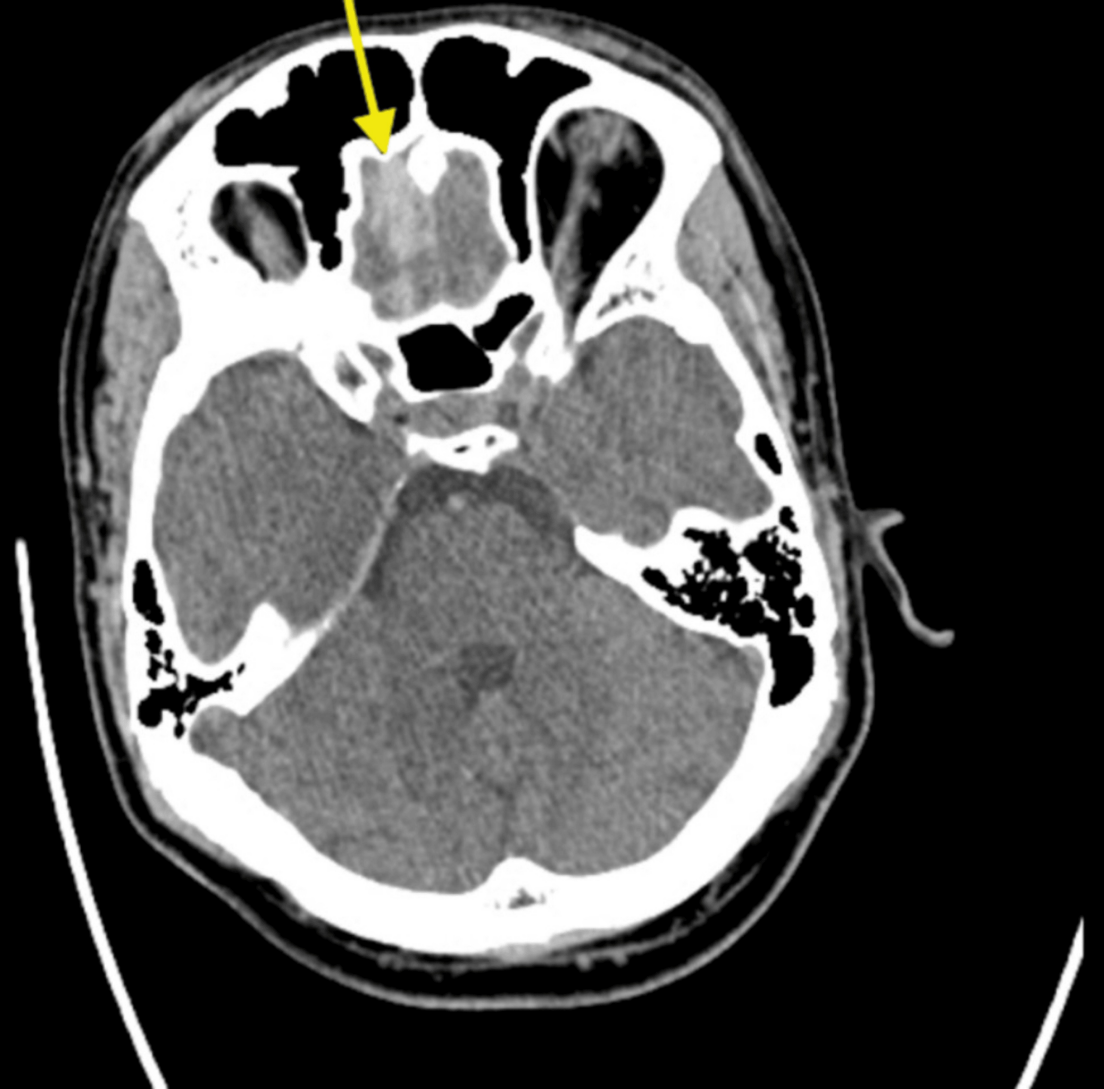
Brain contusion Axial noncontrast CT brain showing a small right frontal brain contusion along the gyrus rectus (yellow arrow). These findings may be compatible with a contrecoup pattern in the appropriate clinical context

Although the patient did not meet guideline-based indications for CT brain imaging, neuroimaging was performed due to persistent headache and clinical concern. Noncontrast CT revealed SAH within the suprasellar cistern, extending along the interhemispheric fissure and inferior frontal cortex (Figure 1), with a small right frontal contusion evident on a higher axial slice (Figure 2). CT angiography (CTA) excluded aneurysm, arteriovenous malformation, or other vascular pathology. The findings were suggestive of a contrecoup-type injury following occipital impact.

The patient was admitted under the Neurosurgery Team and managed conservatively. Treatment included regular neurological observations, analgesia with paracetamol, avoidance of nonsteroidal anti-inflammatory drugs, hydration, and mechanical venous thromboembolism prophylaxis. Nimodipine 60 mg every four hours was prescribed on the recommendation of the neurosurgical team, in line with local practice for cases involving basal cisternal hemorrhage. Although evidence for its use in traumatic SAH is limited, the decision was guided by specialist advice and institutional protocol extrapolated from aneurysmal SAH data, suggesting a potential reduction in delayed cerebral ischemia [6,7]. During admission, his GCS and vital parameters remained stable with no new neurological deficits or worsening headache. He was observed for a couple of days under neurosurgical care before discharge. No surgical intervention was required, and he was discharged with outpatient neurosurgical follow-up.

## Discussion

This case is notable for two reasons. First, it represents a “rule-negative” presentation of traumatic SAH. The patient had a normal GCS, no high-energy trauma, no anticoagulation, and no focal neurological deficits. According to most guidelines, CT brain imaging would not have been indicated [1,2]. However, the persistence of headache after recovery from the syncopal event raised sufficient clinical concern to warrant neuroimaging despite the absence of formal criteria, thereby preventing a potentially missed diagnosis. This highlights the importance of complementing established decision rules with sound clinical judgment.

Second, the mechanism illustrates an uncommon but educational aspect of traumatic brain injury. Contrecoup injuries typically produce cortical contusions in the frontal or temporal lobes after occipital impact [3]. In this case, occipital impact likely caused anterior brain displacement, leading to a right frontal contusion with associated SAH. The hemorrhage pattern in the suprasellar cistern resembled that of an aneurysmal rupture, which complicated initial interpretation.

Traumatic SAH accounts for approximately 33%-60% of all SAH presentations in patients with head trauma [8]. However, contrecoup-associated SAH, particularly those with basal cisternal or aneurysmal-like distributions, remains uncommon in the literature, with only a handful of case reports published to date [3-6]. Sato et al. described basal SAH secondary to cerebellar contrecoup contusions [4], whereas Alhoobi et al. reviewed a decade of nonaneurysmal SAH and emphasized the rarity of trauma-related variants [5]. Of particular relevance, Konczalla et al. analyzed 125 nonaneurysmal, nontraumatic SAH cases and found that nonperimesencephalic hemorrhage patterns, especially those with basal cisternal involvement, were uncommon but clinically significant even when angiography was negative [6]. These findings reinforce that such basal cisternal bleeds warrant close monitoring and CTA to exclude vascular pathology, mirroring the rationale for angiography in our patient despite a clearly traumatic mechanism.

While most cases of traumatic SAH are attributed to cortical vessel injury or contrecoup contusions, CTA should be considered when imaging demonstrates a basal cisternal or perimesencephalic distribution, when the mechanism of injury is minor relative to the hemorrhage burden, or when the clinical presentation appears disproportionate to the apparent trauma. These features may suggest an underlying aneurysmal source that warrants further evaluation. In this case, CTA was performed given the basal cisternal involvement and to exclude vascular pathology, which yielded negative findings [9,10].

This case reinforces the importance of understanding traumatic biomechanics to avoid misdiagnosis and unnecessary aneurysmal workup. It also emphasizes that clinicians should maintain a high index of suspicion and integrate clinical intuition with established imaging guidelines when standard criteria do not fully explain the patient's presentation. Persistent or progressive headache, an atypical mechanism of injury, or disproportionate post-event symptoms should prompt reconsideration of imaging decisions, even in guideline-negative scenarios.

From a clinical standpoint, this case supports including “persistent or unexplained headache following transient loss of consciousness” as a potential imaging trigger within head injury assessment frameworks. Further studies could help refine decision tools such as the NICE Head Injury Guideline and the Canadian CT Head Rule to better capture these low-risk yet clinically significant presentations.

## Conclusions

This case emphasizes that significant intracranial pathology may occur even in patients who do not meet established imaging criteria. Clinical decision rules such as the NICE Head Injury Guideline and the Canadian CT Head Rule are valuable tools, but they are not infallible. Persistent or atypical post-event symptoms-particularly a new, worsening, or unexplained headache that does not resolve after syncope or minor trauma-should prompt reconsideration of neuroimaging, even in guideline-negative patients. Features such as occipital or diffuse headache, associated nausea, or delayed symptom progression may warrant early CT to exclude intracranial bleeding.

Clinicians should therefore complement guideline-based decision-making with careful history-taking, physical examination, and clinical judgment, using established rules as supportive frameworks rather than absolute thresholds. Furthermore, contrecoup injuries, though relatively common, may rarely result in SAH with a distribution mimicking aneurysmal rupture, underscoring the need to interpret imaging findings within the context of the mechanism of injury.

Finally, this case highlights broader implications for current head injury assessment practice: persistent or unexplained headache following transient loss of consciousness may warrant inclusion as a potential “red flag” feature in future iterations of imaging guidelines, helping reduce missed diagnoses in otherwise low-risk patients.
